# The Efficacy of Local Versus Overseas Natural Environments in 360-Degree Virtual Reality Video for Improving Mental Wellness in Medical Students: A Retrospectively Registered Two-Arm Parallel Randomized Trial

**DOI:** 10.3390/healthcare14081087

**Published:** 2026-04-20

**Authors:** Muhammad Hizri bin Hatta, Farah Deena Abdul Samad, Siew Koon Chong, Suriati Mohamed Saini

**Affiliations:** 1 Department of Psychiatry, Faculty of Medicine, Universiti Sultan Zainal Abidin, Medical Campus, Jalan Sultan Mahmud, Kuala Terengganu 20400, Malaysia; hizhatt@gmail.com; 2 Department of Psychiatry, Faculty of Medicine, Universiti Kebangsaan Malaysia, Kuala Lumpur 56000, Malaysia; atisaini@hctm.ukm.edu.my; 3 Department of Psychiatry, Hospital Canselor Tuanku Muhriz, Kuala Lumpur 56000, Malaysia; 4 Jabatan Psikiatri, Hospital Kuala Lumpur, Kuala Lumpur 50300, Malaysia; chongsiewkoon85@gmail.com

**Keywords:** virtual reality, 360-degree VR video, mental wellness, medical student, geographic comparison

## Abstract

**Highlights:**

**What are the main findings?**

- Short, immersive 360-degree VR nature sessions significantly reduce anxiety and stress while improving general well-being in medical students.
- Geographic familiarity (local vs. overseas environments) does not alter the therapeutic effectiveness of these VR sessions.

**What are the implications of the main findings?**

- The restorative psychological effects of virtual nature may be consistent across diverse geographic and cultural contexts.
- Medical schools can affordably support student mental health using standardized, global VR content as a “Digital Green Prescription,” eliminating the need for expensive, localized video production.

**Abstract:**

**Objective:** This study aimed to compare the efficacy of immersive 360-degree Virtual Reality (VR) videos depicting local (Malaysian) versus overseas (Western European) natural environments on the mental health of medical students. The primary outcome was overall mental well-being (WHO-5), and the co-secondary outcomes were changes in anxiety, stress, and depression symptoms (DASS-21). **Methods:** A two-arm parallel randomized trial was conducted with 84 fourth-year and fifth-year medical students. Participants were randomized into two groups (n = 42 each) using a custom, gender-balancing minimization algorithm: Group 1 viewed local environments, and Group 2 viewed overseas environments. Each participant underwent two 15-min VR sessions spaced two weeks apart. Outcomes were measured at baseline (T0), after the first intervention (T1), and at the primary time point after the second intervention (T2). Data were analyzed using a repeated-measures ANOVA with Greenhouse–Geisser and Bonferroni corrections. **Results:** The VR intervention demonstrated a statistically significant improvement in well-being (*p* < 0.001, $\eta_{p}^{2}$ = 0.380) and a significant reduction in anxiety (*p* < 0.001, $\eta_{p}^{2}$ = 0.255) and stress (*p* < 0.001, $\eta_{p}^{2}$ = 0.311) across all participants over time. No significant change was observed in depression scores (*p* = 0.122, $\eta_{p}^{2}$ = 0.028). Notably, there were no statistically significant differences between the local and overseas groups for well-being (*p* = 0.399, $\eta_{p}^{2}$ = 0.011), anxiety (*p* = 0.593, $\eta_{p}^{2}$ = 0.005), stress (*p* = 0.945, $\eta_{p}^{2}$ < 0.001), or depression (*p* = 0.546, $\eta_{p}^{2}$ = 0.006). **Conclusions:** A two-session immersive VR nature intervention is effective for improving well-being and reducing anxiety and stress in medical students. The geographical familiarity of the environment did not significantly impact therapeutic effectiveness, suggesting that the restorative effects of virtual nature may generalize across different environmental and cultural contexts. Trial Registration: NCT07447310; retrospectively registered on 25 February 2026.

## 1. Introduction

The period of medical education is widely recognized as one of the most challenging phases in professional training. Students are subjected to a rigorous curriculum, high academic expectations, and significant emotional and physical demands, which collectively contribute to a high prevalence of psychological distress [1,2]. Research consistently shows that medical students report higher levels of stress, anxiety, and depression compared to the general population and their non-medical peers [3]. This sustained psychological burden not only jeopardizes students’ academic performance and professional development but also poses a significant risk to their long-term mental and physical health. Consequently, the development of accessible, effective, and scalable interventions to support student mental wellness is a critical priority for educational institutions.

In recent years, 360-degree VR video has emerged as a particularly promising and accessible modality for mental healthcare. Unlike computer-generated imagery (CGI) VR, 360-degree videos capture real-world environments, offering a high degree of realism that can enhance the psychological sense of “presence”; the feeling of actually being in the virtual space [4]. This technology has been successfully leveraged for various therapeutic applications. For instance, immersive 360-degree videos of natural scenes have been shown to reduce anxiety in palliative care patients [5] and have been explored as a tool for relaxation and stress reduction in various clinical and non-clinical settings [6,7]. The capacity of 360-degree VR to provide safe, engaging, and realistic simulations makes it an ideal platform for delivering psychological interventions.

The use of natural environments in these interventions is supported by a robust body of evidence on the mental health benefits of nature exposure. A key theoretical framework in this area is Attention Restoration Theory (ART), which suggests that natural environments promote recovery from mental fatigue by engaging involuntary attention, thus allowing directed-attention mechanisms to rest and replenish [8]. While the theory is influential, meta-analytic reviews have shown mixed support for its broader cognitive benefits, with significant effects found for some measures of attention but not for others [9]. Nonetheless, direct exposure to nature has been consistently shown to reduce stress, decrease rumination, and improve mood [10,11]. VR nature environments offer a unique opportunity to “transport” individuals to these restorative settings, delivering the psychological benefits of nature to those who may have limited access due to urbanization, mobility issues, or time constraints [12].

A recent systematic review [13] suggests that virtual reality nature interventions may reduce anxiety and stress among higher education students. However, the review highlighted a notable gap in the current literature: a relative lack of research comparing the therapeutic effects of different types of virtual environments. The present study seeks to address this gap by directly comparing the therapeutic efficacy of familiar (local) versus novel (overseas) virtual natural landscapes to determine if geographic familiarity alters restorative outcomes.

Despite the growing use of VR nature interventions, a significant research gap exists regarding the optimal content of these virtual environments. It remains unclear whether the cultural and geographical familiarity of the environment influences its therapeutic impact. It could be hypothesized that familiar, local environments might evoke a stronger positive response due to personal resonance and a deeper sense of connection. Conversely, a novel, overseas environment could be more effective by providing a greater sense of escapism and mental distance from current stressors. To our knowledge, no prior studies have directly compared the psychological effects of local, familiar versus non-local, unfamiliar virtual natural environments within a single population. This study was therefore designed to address this gap by comparing the effects of local (Malaysian) and overseas (Western European) natural VR environments on the mental health of Malaysian medical students.

## 2. Methods

### 2.1. Study Design and Ethical Approval

This study received ethical approval from the UKM Research Ethics Secretariat (JEPUKM) under reference number JEP-2024-039. The research was conducted as a two-arm parallel randomized controlled trial. A total of 88 participants were initially randomized into the trial. Following recruitment, four participants became unresponsive to follow-up attempts and were excluded, resulting in a negligible overall attrition rate of 4.5%. All remaining participants provided written informed consent.

To minimize bias, the study employed outcome assessor blinding. While the nature of the VR intervention prevented the blinding of participants and the research assistant, the primary investigator responsible for data tabulation and statistical analysis remained fully blinded to group allocation until the final comparative analysis was complete.

### 2.2. Sample Size and Power Analysis

An a priori power analysis was conducted using G*Power software (version 3.1) to determine the minimum sample size required to test the primary hypotheses. The calculation was based on a repeated-measures ANOVA (within-between interaction) to compare two groups across three time points. To ensure the study was adequately powered to detect a small-to-medium effect size, we set the anticipated effect size to Cohen’s *f* = 0.15. Assuming an alpha level of 0.05, a statistical power of 80%, and a conservative correlation among repeated measures of 0.5, the minimum required sample size was calculated to be 72 participants. To account for potential attrition, the recruitment target was increased to 88.

### 2.3. Randomization and Allocation Concealment

Participants were randomly assigned (1:1 ratio) to either Group 1 (Local Environment, n = 42) or Group 2 (Overseas Environment, n = 42). Sequence generation and group allocation were executed using a custom Python 3.13 script utilizing a pseudorandom number generator. To ensure strict allocation concealment, the assignment was performed in real-time by a research assistant as each participant arrived at the trial room for their baseline session. The script utilized a dynamic allocation algorithm (minimization) to randomly assign participants while continuously balancing the male-to-female ratio across groups.

### 2.4. Trial Registration and Transparency

This randomized controlled trial was retrospectively registered with the ClinicalTrials.gov (ID: NCT07447310). The trial was registered on 25 February 2026, which occurred after the commencement of participant recruitment. This retrospective registration was due to an administrative oversight regarding the timing of trial registration at the study’s inception. However, the study protocol, eligibility criteria, and predefined primary and secondary outcomes were established prior to the recruitment of the first participant and remained unchanged throughout the conduct of the study. No modifications were made to the study design or outcome measures during the trial. The trial was subsequently registered to ensure transparency and to align with current clinical trial reporting standards.

### 2.5. Intervention

The intervention consisted of two 15-min sessions of viewing immersive 360-degree VR videos of natural sceneries, with the sessions separated by a two-week interval. The VR content was delivered through Meta Quest 3 VR headsets (Meta Platforms, Inc., Menlo Park, CA, USA). To facilitate a fully immersive experience of the 360-degree environment, participants were seated in a swivel chair, allowing them to freely turn and view their surroundings. To ensure replicability, the 360-degree videos were captured in 5.7 K resolution at 30 frames per second using an Insta360 X3 360-degree action camera (Arashi Vision Inc., Shenzhen, China), accompanied by native audio with no additional audio such as background music.

The 360-degree videos were captured by the primary researcher to ensure consistency in quality and style. The content was designed to have directly comparable scenes between the two groups. Within each 15-min session, participants were exposed to a sequence of all four scenery types for that condition, with each scene lasting approximately 3 min and 45 s before transitioning to the next.

The four types of natural scenery were:

1. Forest: Malaysian rainforest versus a temperate Western European forest.
2. Beach: A tropical Malaysian beach with palm trees versus a Western European beach.
3. River: A calm local river scene flanked by tropical vegetation versus a European river scene
4. City Park: A public park located in Kuala Lumpur versus a public park in a European city.

Screenshots of each environment and their counterparts can be viewed in the Supplementary Materials.

### 2.6. Outcome Measures

Psychological outcomes were assessed at three distinct time points: before the first intervention (T0, baseline), after the first intervention (T1), and after the second intervention (T2). The following validated self-report questionnaires were administered:

#### 2.6.1. Primary Outcome Measure

WHO-5 Well-Being Index (WHO-5): The WHO-5 is a brief, 5-item self-report measure of subjective psychological well-being. It was developed by the World Health Organization and has been validated as a sensitive and specific screening tool for depression and a reliable measure of well-being [14]. T2 was designated as the primary time point of interest as it aligns with the two-week recall window of the WHO-5. Participants rate each item on a 6-point scale from 0 (“At no time”) to 5 (“All of the time”) based on their feelings over the preceding two weeks. The raw score is calculated by summing the responses (ranging from 0–25). This raw score is then multiplied by 4 to convert it to a percentage scale (0–100), where higher scores reflect better well-being.

#### 2.6.2. Secondary Outcome Measure

Depression, Anxiety and Stress Scale—21 Items (DASS-21): The DASS-21 subscales served as co-secondary outcomes. The DASS-21 is a widely used and well-validated instrument designed to measure the severity of the core symptoms of depression, anxiety, and stress [15]. It consists of 21 items, with 7 items per subscale. Participants rate the extent to which they have experienced each state over the past week on a 4-point Likert scale. The validity and reliability of the DASS-21 have been established across numerous populations, including Malaysian samples [16]. Subscale scores are calculated by summing the scores for the relevant items and multiplying the result by two, as per the official scoring manual. Higher scores indicate greater severity of symptoms.

### 2.7. Statistical Analysis

Data were analyzed using IBM SPSS software (Version 31). A repeated-measures analysis of variance (ANOVA) was employed to assess the main effect of time and the interaction effect of time and group. Because missing data was not imputed, all analyses were conducted on a per-protocol basis for the 84 participants who completed all assessments.

The assumption of sphericity was assessed using Mauchly’s test; where violated, degrees of freedom were corrected using Greenhouse–Geisser estimates. Post hoc pairwise comparisons were conducted using Bonferroni corrections to control for multiple comparisons. Statistical significance was set at *p* < 0.05 (two-tailed), and results are reported with exact *p*-values (including leading zeros), 95% confidence intervals (CIs), and partial eta-squared ($\eta_{p}^{2}$) effect sizes.

## 3. Results

**Figure 1:**
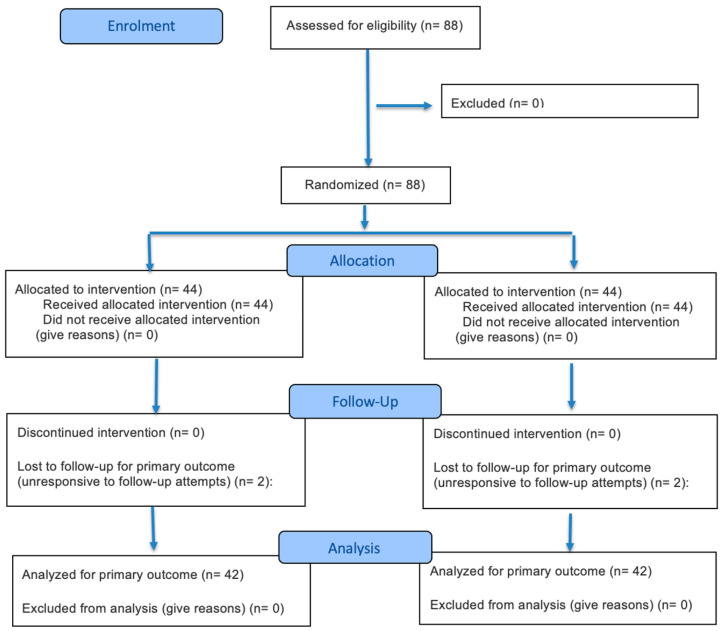
CONSORT 2025 Participant Flow Diagram.

The flow of participants through the trial, including enrollment, randomization, follow-up, and data analysis, is detailed in the CONSORT 2025 flow diagram (Figure 1) below.

### 3.1. Overall Effect of VR Intervention Regardless of Group

The analysis first examined the overall effect of the VR intervention on all participants, regardless of their assigned group. The results indicated that the intervention had a significant positive impact on the primary and several secondary measures of mental health.

#### 3.1.1. Primary Outcome: Well-Being (WHO-5)

**Figure 2:**
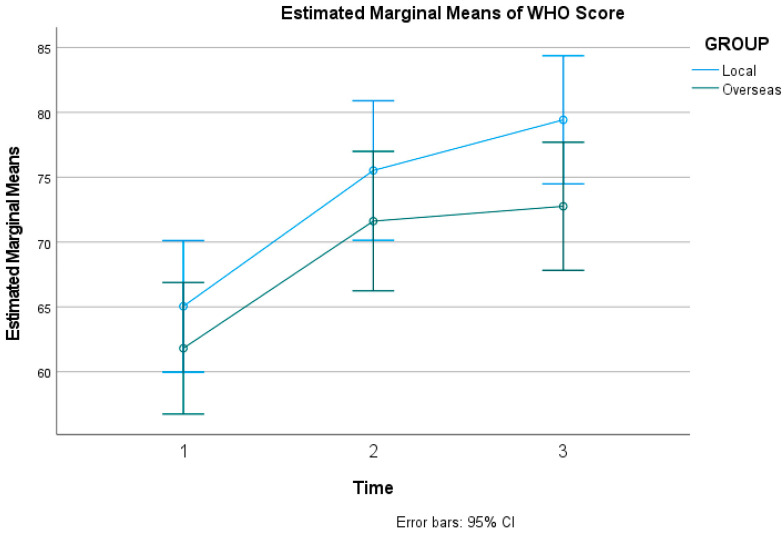
Estimated Marginal Means of WHO-5 score.

The intervention led to a significant improvement in participants’ overall sense of well-being. Mauchly’s test indicated that the assumption of sphericity was met for well-being scores ($\chi^{2}(2)$ = 4.42, *p* = 0.110). The analysis confirmed a statistically significant increase in WHO-5 scores over time (*F*(2, 164) = 50.22, *p* < 0.001, $\eta_{p}^{2}$ = 0.380), indicating that participants felt significantly better after the VR sessions. The grand mean score for WHO-5 across all time points and participants was 71.032, with a standard error (SE) of 1.653, and a 95% confidence interval (CI) with a lower bound of 67.743 and an upper bound of 74.320. Figure 2 below shows the estimated marginal means of the WHO-5 score.

#### 3.1.2. Secondary Outcome: Anxiety

**Figure 3:**
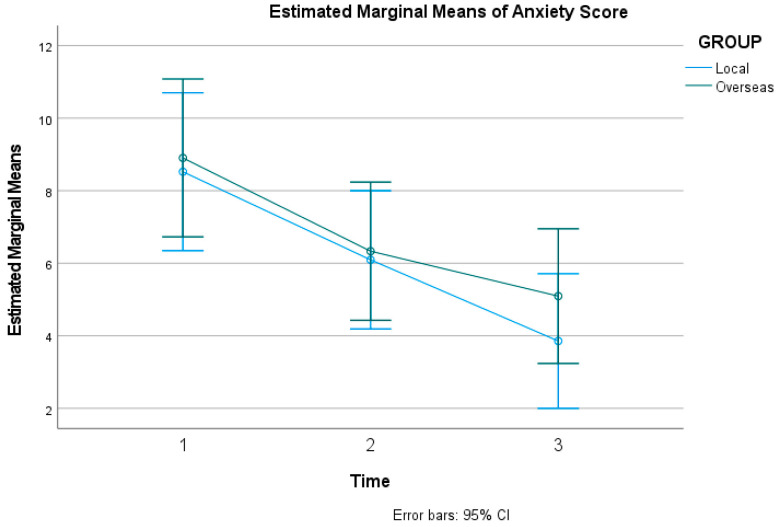
Estimated Marginal Means of DASS-21 for anxiety score.

Participants experienced a statistically significant reduction in anxiety levels across the duration of the study in both groups. Mauchly’s test indicated that the assumption of sphericity was violated for anxiety scores ($\chi^{2}(2)$ = 24.45, *p* < 0.001); therefore, degrees of freedom were corrected using Greenhouse–Geisser estimates. The repeated-measures ANOVA revealed a statistically significant main effect of time on DASS-21 anxiety scores (*F*(1.59, 130.10) = 28.00, *p* < 0.001, $\eta_{p}^{2}$ = 0.255). The grand mean score for anxiety across all time points and participants was 6.468, with a standard error (SE) of 0.624, and a 95% confidence interval (CI) with a lower bound of 5.227 and an upper bound of 7.709. Figure 3 below shows the estimated marginal means of the DASS-21 for anxiety score.

#### 3.1.3. Secondary Outcome: Stress

**Figure 4:**
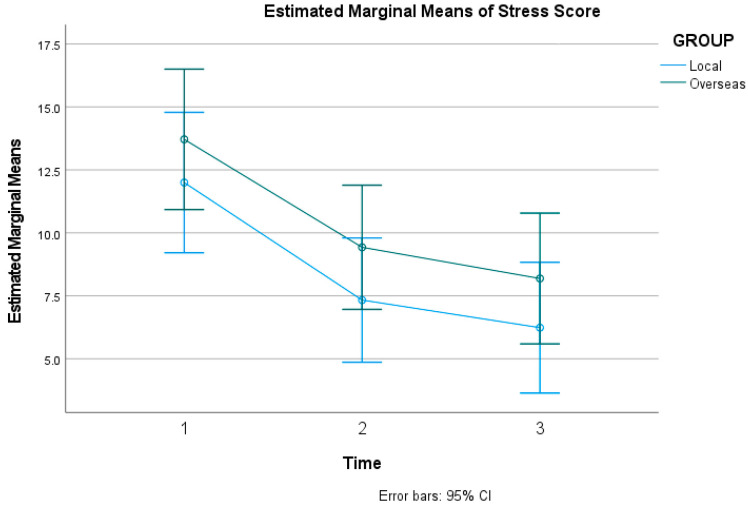
Estimated Marginal Means of DASS-21 for stress score.

A similar positive outcome was observed for stress. Mauchly’s test indicated that the assumption of sphericity was violated for stress scores ($\chi^{2}$(2) = 14.26, *p* < 0.001); therefore, degrees of freedom were corrected using Greenhouse–Geisser estimates. The results showed a statistically significant decrease in participants’ reported stress levels from baseline to the final measurement point, confirmed by a significant main effect of time on DASS-21 stress scores (*F*(1.72, 141.21) = 36.95, *p* < 0.001, $\eta_{p}^{2}$ = 0.311). The grand mean score for stress across all time points and participants was 9.484, with a standard error (SE) of 0.841, and a 95% confidence interval (CI) with a lower bound of 7.811 and an upper bound of 11.157. Figure 4 below shows the estimated marginal means of the DASS-21 for stress score.

#### 3.1.4. Secondary Outcome: Depression

**Figure 5:**
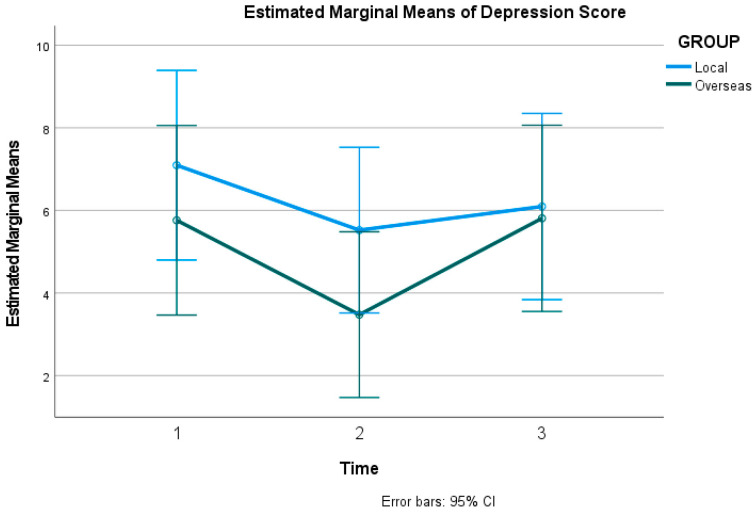
Estimated Marginal Means of DASS-21 depression score.

In contrast to well-being, anxiety, and stress, the intervention did not have a significant impact on symptoms of depression. Mauchly’s test of sphericity indicated that the assumption of sphericity was violated for depression scores ($\chi^{2}$(2) = 71.38, *p* < 0.001); therefore, Greenhouse–Geisser corrected estimates are reported. The analysis showed no statistically significant change in DASS-21 depression scores over the three time points (*F*(1.26, 103.42) = 2.34, *p* = 0.122, $\eta_{p}^{2}$ = 0.028). The grand mean score for depression across all time points and participants was 5.627, with a standard error (SE) of 0.564, and a 95% confidence interval (CI) with a lower bound of 4.505 and an upper bound of 6.749. Figure 5 below shows the estimated marginal means of the DASS-21 for depression score.

### 3.2. Comparison of Local vs. Overseas Environments

The central aim of the study was to determine if the geographical setting of the VR environment influenced its effectiveness. The analysis of the interaction between time and group found no significant differences between the participants who viewed local scenes and those who viewed overseas scenes.

#### 3.2.1. Primary Outcome (WHO-5) Interaction

The improvement in well-being was comparable across both groups. The analysis showed no significant difference in the change in WHO-5 scores over time between the local and overseas conditions (*F*(2, 164) = 0.92, *p* = 0.399, $\eta_{p}^{2}$ = 0.011).

#### 3.2.2. Secondary Outcomes (DASS-21) Interaction

The effectiveness of the intervention in reducing negative emotional states did not differ between the two groups. The interaction effect between time and environmental group was not statistically significant for Anxiety (*F*(1.59, 130.10) = 0.45, *p* = 0.593, $\eta_{p}^{2}$ = 0.005), Stress (*F*(1.72, 141.21) = 0.04, *p* = 0.945, $\eta_{p}^{2}$ < 0.001), or Depression (*F*(1.26, 103.42) = 0.46, *p* = 0.546, $\eta_{p}^{2}$ = 0.006).

In summary, these results indicate that while the two-session VR nature intervention was effective for improving well-being and reducing anxiety and stress, its therapeutic power was not dependent on whether the depicted natural environment was local or foreign to the participant.

## 4. Discussion

This study provides compelling evidence that a multi-session intervention using immersive 360-degree VR nature videos can significantly reduce symptoms of anxiety and stress and improve subjective well-being among medical students. The observed improvements in anxiety and stress are consistent with the principles of Attention Restoration Theory [8], which posits that natural environments can replenish directed attention and reduce mental fatigue, although the broader cognitive benefits of ART have received mixed support in meta-analytic reviews [9]. Our findings contribute to a growing body of evidence supporting the use of VR nature exposure as an effective, non-pharmacological tool for mental health management [17,18].

The primary and most novel finding of this study, however, was the lack of a significant difference in efficacy between local Malaysian and overseas Western European natural scenes. This suggests that the therapeutic benefits of virtual nature may generalize across different environmental and cultural contexts. This outcome may be primarily driven by the immersive quality of the VR experience itself. The technology’s ability to induce a strong sense of presence may provide a powerful feeling of escapism, temporarily transporting the individual away from their immediate stressors [4]. In this context, the act of “being away” may be more therapeutically important than the specific content of “where” one is. Furthermore, it is possible that core restorative elements of nature (such as lush greenery, the presence of water, and open vistas) are broadly recognized by the human brain as calming and restorative, regardless of their specific geographical location.

It is noteworthy that the intervention did not produce a statistically significant reduction in depression scores. This result is not entirely unexpected and may be attributable to several factors. First, the baseline depression scores of the participant sample were, on average, within the normal-to-mild range on the DASS-21, creating a potential floor effect where significant further reduction is difficult to achieve or measure. Second, depressive symptoms, which are often characterized by persistent low mood and anhedonia, may be more trait-like and resilient to short-term interventions compared to more state-like conditions of acute anxiety and stress [19]. It is plausible that alleviating depressive symptoms would require interventions of a longer duration, higher frequency, or those that more directly target cognitive patterns, rather than relying on passive environmental exposure.

From a public health and institutional perspective, the lack of geographical preference offers a major economic advantage. Developing high-quality, localized VR content is resource-intensive. Our findings suggest that medical schools can utilize standardized, global VR nature libraries to achieve maximum therapeutic return of investment (ROI) [12]. For urban medical campuses where physical green space is either scarce or cost-prohibitive to develop, a “Digital Green Prescription” offers a pragmatic, space-efficient alternative. By integrating these 15-min VR sessions into the existing medical curriculum, universities can provide a scalable intervention to combat the “hidden curriculum” of burnout. This approach essentially democratizes access to nature’s restorative benefits, providing a consistent mental health resource regardless of a campus’s physical location or surrounding infrastructure.

### 4.1. Limitations

Despite its novel findings, this study has several limitations that should be considered. First, the sample was homogenous, drawn from a single institution and consisting exclusively of medical students. This limits the generalizability of the findings to other demographic groups, such as non-student populations or individuals from different cultural backgrounds. Second, the study design lacked a no-intervention control group. While the pre-intervention baseline (T0) served as a robust within-subject control, the absence of a passive control group makes it difficult to definitively separate the effects of the VR intervention from potential confounding factors such as the placebo effect or demand characteristics. Third, there is an inherent discrepancy between the retrospective recall windows of the psychological scales (one week for the DASS-21, two weeks for the WHO-5) and the immediate post-intervention assessment at T1. Consequently, T1 scores may reflect immediate “state-like” mood shifts rather than true retrospective changes. Finally, the absence of statistically significant differences between the local and overseas groups should not be definitively interpreted as proof of equivalence. Without formal equivalence testing, it is not possible to conclusively rule out meaningfully large differences between the environmental conditions.

### 4.2. Future Directions

Building on these findings, future research could proceed in several promising directions. A three-arm randomized controlled trial that includes a no-intervention or sham-VR control group would be essential to further validate the efficacy of the VR nature intervention. Longitudinal studies are needed to determine the long-term effects and to explore optimal “dosing,” including the ideal length, frequency, and number of sessions required for sustained improvement. Additionally, future studies assessing immediate post-intervention effects should utilize specific “state” measures (e.g., STAI-State) rather than retrospective trait-oriented scales. Future studies could also incorporate physiological measures, such as heart rate variability (HRV) and salivary cortisol, to provide objective biomarkers of stress reduction. Furthermore, researchers comparing different VR environments should employ formal equivalence analysis, such as Two One-Sided Tests (TOST) or Bayesian methods, to statistically demonstrate therapeutic equivalence. Finally, investigating participant preferences and allowing for the personalization of virtual environments could provide valuable insights into enhancing engagement and maximizing the therapeutic potential of VR for mental wellness.

## 5. Conclusions

Immersive 360-degree VR nature videos represent a promising and effective modality for the enhancement of general mental well-being and the reduction in anxiety and stress in a high-stress academic population. The effectiveness of this intervention does not appear to be dependent on the geographical familiarity of the virtual environment, whether depicting familiar local natural landscapes or less familiar overseas environments. This suggests that the restorative power of nature, when delivered virtually, may generalize across different environmental and cultural contexts, offering a scalable and accessible mental health tool.

## Data Availability

The datasets generated and analyzed during the current study, alongside the custom Python randomization script and statistical analysis protocols, are not publicly available to protect participant privacy and maintain data confidentiality as per the ethical approval guidelines (JEP-2024-039). However, de-identified datasets and codes are available from the corresponding author upon reasonable request. Detailed technical characteristics of the VR intervention are described in Section 2, and visual representations of the environments are available in the Supplementary Materials.

## Supplementary Materials

The following supporting information can be downloaded at: https://www.mdpi.com/article/10.3390/healthcare14081087/s1, Comparable photos of the four types of natural scenery.
