# Supplementary material for: The Efficacy of Local Versus Overseas Natural Environments in 360-Degree Virtual Reality Video for Improving Mental Wellness in Medical Students: A Retrospectively Registered Two-Arm Parallel Randomized Trial
Source: Healthcare (Basel). 2026 Apr 20;14(8):1087. doi: 10.3390/healthcare14081087 (PMC13115815; doi:10.3390/healthcare14081087)
Supplement: Supplementary file 1 [file healthcare-14-01087-s001.zip › healthcare-4190443-supplementary.pdf]

Supplemental Materials

Comparable photos of the four types of natural scenery:

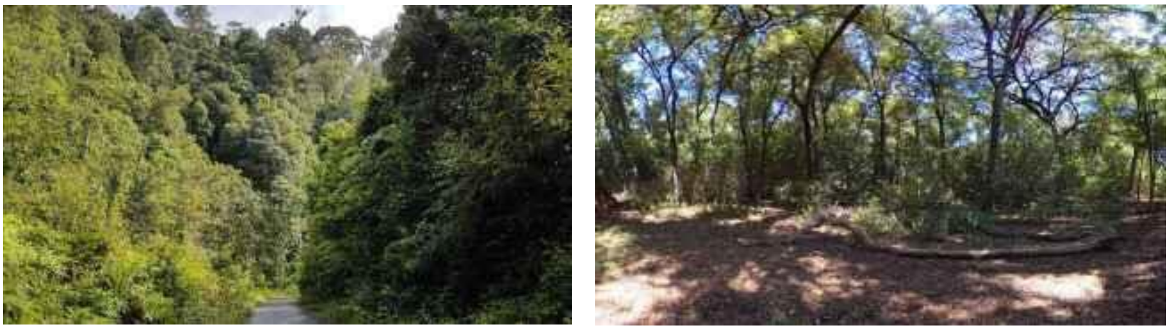

Forest: Malaysian rainforest (left) versus a temperate Western European forest (right).

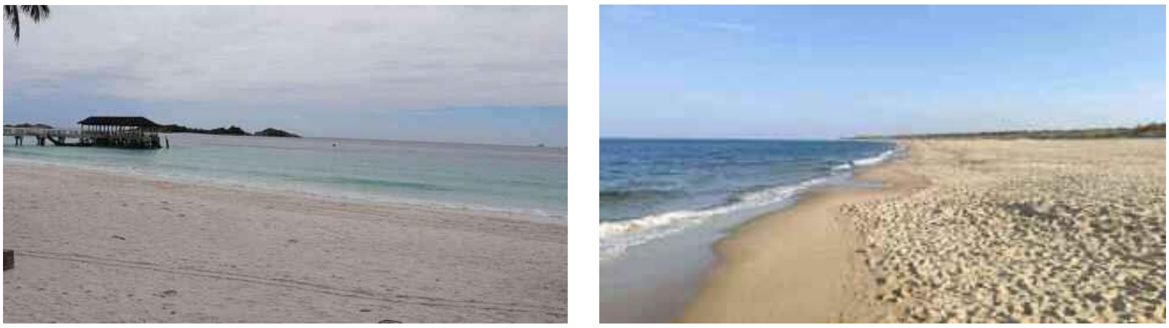

Beach: A tropical Malaysian beach with palm trees versus a Western European beach.

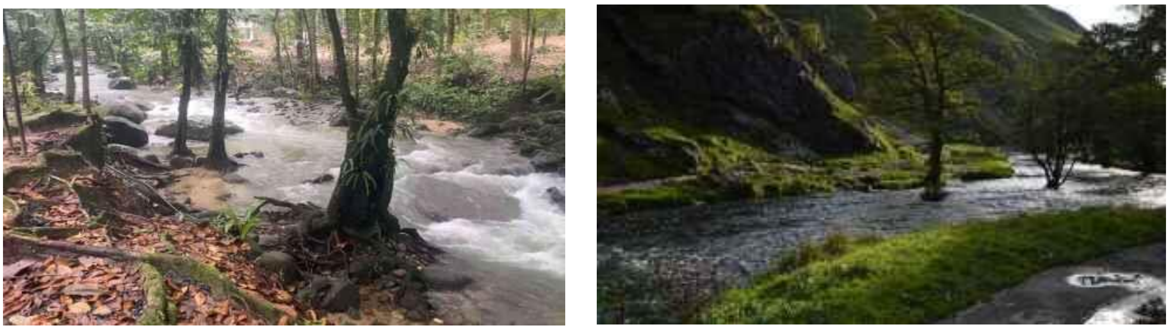

River: A calm local river scene flanked by tropical vegetation versus a European river scene.

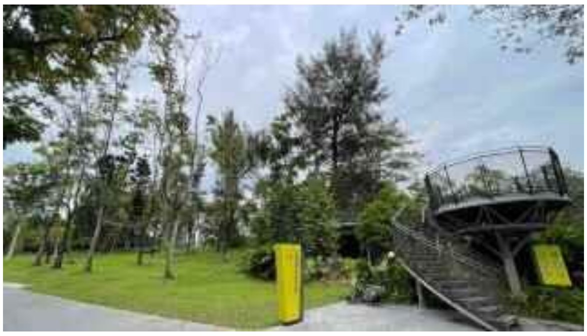 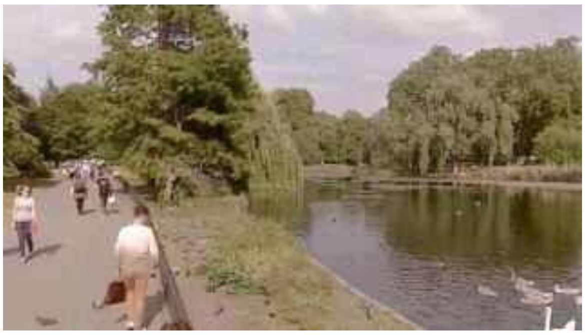

City Park: A public park located in Kuala Lumpur versus a public park in a European city.
